# Proximal Humerus Fracture/Dislocation: Look for the Greater Tuberosity

**DOI:** 10.7759/cureus.33795

**Published:** 2023-01-15

**Authors:** Delaram Mostowfi Zadeh, Karim Abdelghafour, Angelos Assiotis

**Affiliations:** 1 Trauma and Orthopaedics, Lister Hospital, Stevenage, GBR

**Keywords:** pre-contoured locking plate, computerized tomography scan, missed fracture, greater tuberosity, shoulder fracture dislocation

## Abstract

The shoulder is the commonest major joint involved in dislocations. These are often associated with fractures of the surgical neck and/or of the greater tuberosity of the proximal humerus. A good functional recovery is associated with a successful union of the tuberosity fragment, as this carries the insertion of the superior and posterior rotator cuff tendons. A 29-year-old male patient presented to our Emergency Department (ED) after a fall off his motorbike, resulting in a left shoulder fracture dislocation and an axillary nerve injury. His shoulder was reduced under sedation in the ED, with post-reduction radiographs demonstrating a seemingly satisfactory fracture position. Later on, a computerized tomography (CT) scan was arranged which actually confirmed significant displacement of his greater tuberosity, which was not picked up on initial post-reduction radiographs. As a result of identifying the displacement, surgical fixation with a locking plate and suture construct was undertaken. This case demonstrates the ease with which greater tuberosity fractures can mistakenly be presumed as reduced on post-reduction films, whilst in fact they can be significantly displaced. This risk is especially great when only one radiographic view is obtained. The sign of the ‘disappearing tuberosity’ on a plain radiograph should prompt the clinician to seek further imaging by way of CT, to uncover the true position of the greater tuberosity.

## Introduction

Proximal humerus fractures and shoulder dislocations are fairly prevalent traumatic injuries, particularly in the context of high-risk sports such as motorbike racing, where 47.5% of accidents can result in upper extremity injury [1]. Greater tuberosity fractures may occur in up to 20% of those cases presenting with anterior shoulder dislocations. Surgical fixation is indicated where significant displacement is present, always considering the patient's age and level of activity [2]. Union of the greater tuberosity in an anatomical or near-anatomical position is a crucial prognostic factor for the function of the shoulder because the tuberosity carries the insertions of the supraspinatus, infraspinatus, and teres minor tendons. As such, non-unions or malunions of greater tuberosity fragments result in the lack of active elevation and external rotation, as the rotator cuff tendons are de-tensioned and function at a mechanical disadvantage. There is often also bony impingement under the acromion, if the tuberosity migrates superiorly and with the posterior glenoid, if the tuberosity migrates posteriorly. The main issue with a malunited or non-united greater tuberosity fracture is that the salvage options, such as osteotomy and the tuberosity and/or shoulder arthroplasty surgery, usually yield poor functional outcomes [3]. It is therefore imperative to diagnose such injuries early on after the presentation and to treat them with reduction and fixation.

## Case presentation

**Figure 1:**
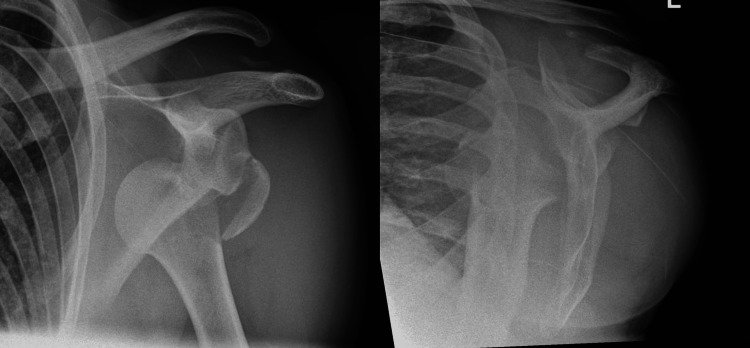
Antero-posterior and lateral views of left shoulder on presentation Demonstrating an anterior dislocation of the humeral head and a displaced fracture of the greater tuberosity. There is also evidence of an acromio-clavicular injury, which was old and not relevant to the current presentation.

A young male patient was referred to our Emergency Department (ED) after a fall off his motorcycle at 30mph whilst racing on a track, resulting in a left non-dominant shoulder fracture dislocation (Figure 1).

**Figure 2:**
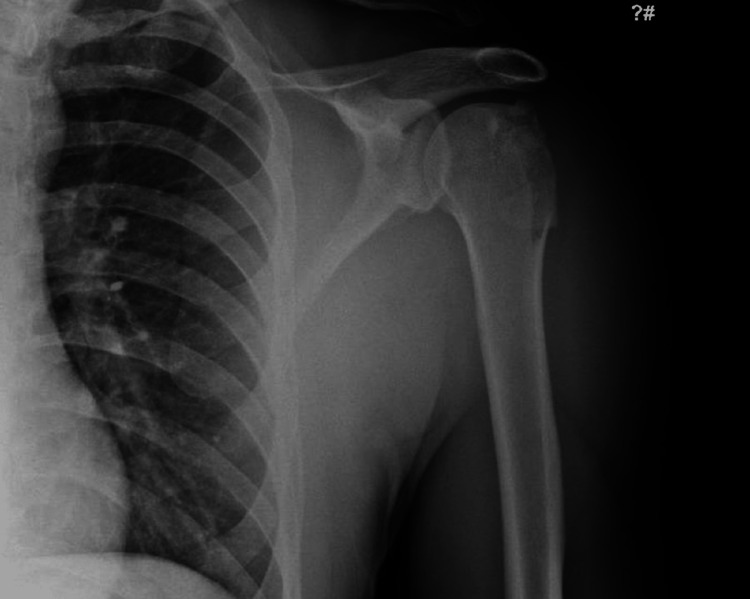
Post-reduction anteroposterior radiograph Demonstrating a successful reduction of the shoulder joint. Note that the greater tuberosity is not so well defined on this view.

He also had reduced sensation in the regimental badge area, indicative of an axillary nerve injury. There was no further neurovascular deficit. His shoulder was reduced under sedation in the ED, with post-reduction radiographs reviewed and accepted as demonstrating a successful joint reduction (Figure 2). Given the deemed satisfactory appearance of the shoulder, the patient was referred routinely through our virtual fracture clinic pathway.

**Figure 3:**
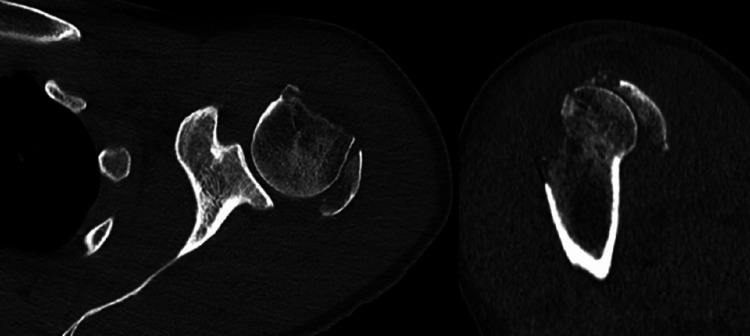
CT scan axial and sagittal images, obtained on presentation to the upper limb fracture clinic These demonstrate significant displacement of the greater tuberosity fragment in a posterior and superior direction, relevant to the humeral head. Healing in such a position, the patient would experience a mechanical block to external rotation and significant weakness, secondary to mechanical disadvantage of the rotator cuff tendons. CT: Computerized tomography

**Figure 4:**
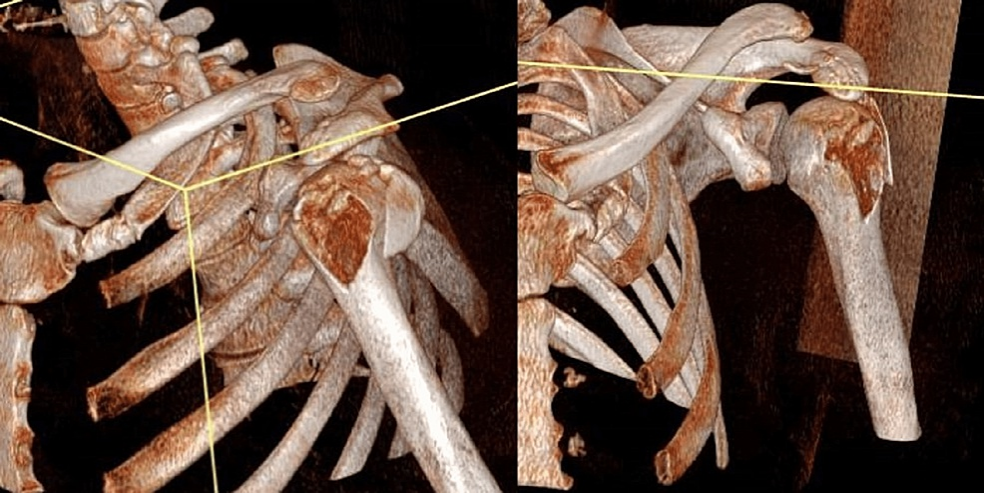
Three-dimensional reconstruction images from the patient's CT scan These give a better impression of the significant displacement of the greater tuberosity fragment and serve to demonstrate how deceptive plain radiographs can be, if not interpreted carefully and with a high index of suspicion. CT: Computerized tomography

He was subsequently reviewed in our Upper Limb trauma service, where a computerized tomography (CT) scan was arranged (Figure 3 and Figure 4).

**Figure 5:**
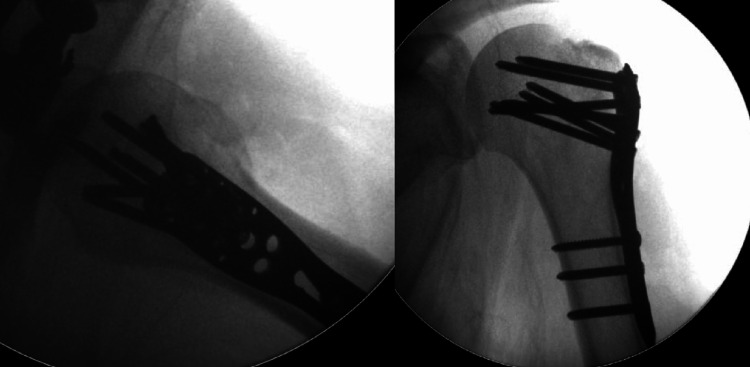
Intra-operative images from the surgical fixation procedure These demonstrate anatomical reduction of the greater tuberosity fragment, held in place with sutures and a pre-contoured fixed-angle locking plate.

This confirmed significant posterior displacement of his greater tuberosity. This displacement was present on the post-reduction plain radiograph but was not recognized. As a result of identifying the displacement, surgical fixation with a locking plate and suture construct was undertaken through a deltoid-splitting approach, seven days following presentation to the ED (Figure 5). The sutures used were size 2 FiberWire sutures, and they were passed through the k-wire holes of the plate.

We decided to use this approach to the fracture as it gives us greater access to posteriorly displaced greater tuberosity fragments and allows for better reduction and fixation, regardless of the chosen mode of fixation. On this occasion, we selected sutures as the primary mode of fixation and augmented the construct with a plate in buttress mode, as the fragment was sizeable and not significantly comminuted.

**Figure 6:**
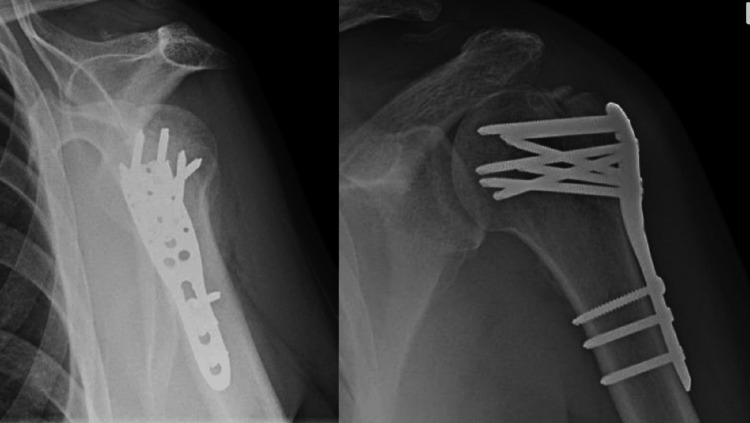
Post-operative antero-posterior and lateral radiographs These demonstrate anatomical reduction of the tuberosity and an advanced degree of union of this fragment.

Post-operative rehabilitation consisted of a sling for six weeks, with onset of physiotherapy immediately after surgery, as per our unit’s protocol for proximal humerus fractures. At two months following his operation, he had no post-operative complication, and there was complete resolution of his axillary nerve symptoms, with improving range of movement. Post-operative radiographs taken at that time demonstrated a satisfactory and anatomical position and alignment of the greater tuberosity fragment (Figure 6).

## Discussion

Shoulder dislocations whether with or without an associated proximal humerus fracture can present with an axillary nerve injury in up to 50% of cases, which is usually a neurapraxia that will eventually recover [4]. It is also common that in the presence of a shoulder dislocation and a greater tuberosity fracture, an additional fracture line in the humeral neck is also present but often not visible on plain radiographs. It has been described that missed humeral surgical neck fractures co-exist in 7.4% of 'isolated' greater tuberosity fractures on a re-review of plain radiographs [5]. For shoulder dislocations associated with greater tuberosity fractures, our unit’s protocol suggests closed reduction of these injuries to take place in an operating theatre, with image intensifier use and in an anaesthetized and muscle-relaxed patient, so as to avoid forceful and ill-controlled reduction maneuvers which may result in significant displacement of a previously undisplaced humeral neck fracture.

This case does not aim to present a rare or unique pathology; instead, it is dealing with a fairly common injury that is also often underdiagnosed and mismanaged, in many healthcare settings. We aim to raise awareness on the diagnosis and appropriate prompt management. This case demonstrates the ease with which greater tuberosity fractures can mistakenly be presumed to be reduced on single post-manipulation radiographs, whilst in fact being significantly displaced, with the fragment actually lying behind the humeral head. It is very easy to mistakenly consider such a radiograph as showing a good position for the fracture to unite in, especially when only one view is taken. The sign of the ‘disappearing tuberosity’ should alert clinicians that the fragment is in fact greatly displaced and lies behind the humeral head. In such cases, a CT scan is required, which will demonstrate the true position of the greater tuberosity.

## Conclusions

Clinicians should actively look for the greater tuberosity fragment on the post-reduction films and, if this is missing, obtain an urgent CT scan. Patients with a ‘disappearing tuberosity’ should be urgently referred to a fracture clinic with an upper limb specialization, if available, as their treatment will likely be surgical. Outcomes for such injuries are time-critical. Hence, there is a need for timely intervention when this is indicated. Finally, reduction of shoulder fracture/dislocations should ideally be done in an operating theatre rather than the ED, in order to avoid displacing an undiagnosed surgical neck fracture that sometimes co-exists with greater tuberosity fractures of the proximal humerus.
